# Draft Genome Sequence of the Pathogenic Filamentous Fungus *Aspergillus lentulus* IFM 54703^T^

**DOI:** 10.1128/genomeA.01568-15

**Published:** 2016-01-14

**Authors:** Yoko Kusuya, Kanae Sakai, Katsuhiko Kamei, Hiroki Takahashi, Takashi Yaguchi

**Affiliations:** a Medical Mycology Research Center, Chiba University, Chiba, Japan; b Molecular Chirality Research Center, Chiba University, Chiba, Japan

## Abstract

*Aspergillus lentulus*, a sibling species of *Aspergillus fumigatus*, has been reported as a causative agent of aspergillosis, and exhibited low susceptibility to azole. Here, we present the draft genome sequence of *A. lentulus* strain IFM 54703^T^ for the first time.

## GENOME ANNOUNCEMENT

Aspergillosis is a clinically important mycosis that comprises a wide variety of bronchopulmonary infections, such as invasive pulmonary aspergillosis, fungus ball in the lung cavity, and allergic bronchopulmonary aspergillosis (1). The most significant causative agent is *Aspergillus fumigatus*. Recently, it has been reported that cases of aspergillosis are caused by closely related species, phylogenetically distinguished with *A. fumigatus*. *Aspergillus lentulus*, one of these sibling species isolated from clinical specimens in the United States was described as a new species, not able to survive at 48°C, and potentially drug resistant, especially to voriconazole (2, 3). The molecular mechanisms of resistance against azole for *A. lentulus* are dependent on 14-α sterol demethylase (Cyp51A) but are different than what has been described previously for *A. fumigatus* (4). The whole-genome sequence of *A. lentulus* may be useful for elucidating the mechanisms resistant to antifungal agents, developing appropriate therapy in aspergillosis, and addressing the genetic diversity of *Aspergillus* species.

The fungal strain used in this study, *A. lentulus* IFM 54703^T^, was stored and maintained at the Medical Mycology Research Center, Chiba University (IFM strains) in Japan. *A. lentulus* genomic DNA extracted from 1-day-old culture by phenol-chloroform extraction and Nucleobond AXG column (TaKaRa) with Nucleobond buffer set III (TaKaRa). Genome sequencing was performed on a Pacific Biosciences RS II (Pacific Biosciences, Menlo Park, CA, USA) using libraries prepared with the SMRTbell template prep kit 1.0 (Pacific Biosciences). A draft genome of *A. lentulus* was assembled using SMRT Analysis 2.3 (Pacific Biosicences) (5). The sequencing runs and assembly of the libraries were carried out by TaKaRa Bio (Mie, Japan). Eventually, 19 scaffolds were obtained, summing up to 30,956,128 bp with an overall G+C content of 49.45%. The *N*_50_ and maximum scaffold were 4,166,724 bp, and 5,062,644 bp, respectively. Gene annotation using AUGUSTUS program 2.5.5 (6) trained with the parameters of the species *Aspergillus fumigatus* resulted in 9,860 genes. 200 tRNAs and 106 rRNAs were predicted by tRNAscan-SE 1.3.1 (7) and RNAmmer 1.2 (8), respectively.

### Nucleotide sequence accession numbers.

The whole-genome sequence of *A. lentulus* has been deposited at NCBI under the accession numbers BCLY01000001 to BCLY01000019.
